# Error estimates of finite element methods for fractional stochastic Navier–Stokes equations

**DOI:** 10.1186/s13660-018-1880-y

**Published:** 2018-10-19

**Authors:** Xiaocui Li, Xiaoyuan Yang

**Affiliations:** 1 School of Science, Beijing University of Chemical Technology, Beijing, P.R. China; 2 LMIB and School of Mathematics and Systems Science, Beihang University, Beijing, P.R. China

**Keywords:** 60N15, 65M60, 60N30, 75D05, Fractional stochastic Navier–Stokes equations, Finite element method, Error estimates, Strong convergence

## Abstract

Based on the Itô’s isometry and the properties of the solution operator defined by the Mittag-Leffler function, this paper gives a detailed numerical analysis of the finite element method for fractional stochastic Navier–Stokes equations driven by white noise. The discretization in space is derived by the finite element method and the time discretization is obtained by the backward Euler scheme. The noise is approximated by using the generalized $L_{2}$-projection operator. Optimal strong convergence error estimates in the $L_{2}$-norm are obtained.

## Introduction

Fractional calculus has been widely used in various applications in science and engineering. It can successfully describe many phenomena in physics, engineering, biology, chemistry, and even economics. Fractional differential equations are more appropriate for the description of memorial and hereditary properties of various materials and processes than the previously used integer order models, and, as a result, a number of numerical techniques for fractional differential equations have been developed and their stability and convergence have been investigated, see, e.g., [1–11]. Besides, many works have been done theoretically or numerically on the stochastic differential equations [12–24].

Fractional Navier–Stokes equations (FNSEs) are widely regarded as some of the most fascinating problems in fluid mechanics, in particular, they could even lead to a better understanding of the physical phenomena and mechanisms of turbulence in fluids [25]. Furthermore, the presence of noises could give rise to some statistical features and important phenomena, for example, a unique invariant measure and ergodic behavior driven by degenerate noise have been established. At the same time, the stochastic perturbations cannot be avoided in a physical system, sometimes they even cannot be ignored. Hence fractional stochastic Navier–Stokes equations have been proposed, which display the behavior of a viscous velocity field of an incompressible liquid and have wide application value in the fields of physics, chemistry, population dynamics, and so on [26–28].

This article is devoted to the study of the error estimates of the finite element method for the incompressible fractional stochastic Navier–Stokes equations

1.1 $$\begin{aligned} \textstyle\begin{cases} u_{t}+\mathscr{B}^{\alpha }\mathscr{L}u+u\cdot \nabla u+\nabla p= \dot{W}, &\text{in } \Omega \times [0, T], \\ \nabla \cdot u=0, &\text{in } \Omega \times [0, T], \\ u(x,0)=u_{0}, &\text{in } \Omega \\ u=0, &\text{on } \partial \Omega \times [0, T], \end{cases}\displaystyle \end{aligned}$$

where $\Omega \subset \mathbf{R}^{2}$ is a bounded and connected polygonal domain, *u* represents the velocity field, *p* is the associated pressure, $u_{0}$ is the initial velocity and the right-hand side term *Ẇ* denotes the white noise, $\mathscr{L}u=-\triangle u$; $\mathscr{B}^{\alpha }:=^{R}D_{t}^{1-\alpha }$ is the Riemann–Liouville fractional derivative in time defined for $0<\alpha <1$ by

1.2 $$\begin{aligned} \mathscr{B}^{\alpha }\varphi (t):=\frac{\partial }{\partial t} \mathscr{I}^{\alpha }\varphi (t):=\frac{\partial }{\partial t} \int _{0}^{t}\omega_{\alpha }(t-s)\varphi (s) \,ds\quad \text{with } \omega_{\alpha }(t):=\frac{t^{\alpha -1}}{\Gamma (\alpha )}, \end{aligned}$$

where $\mathscr{I}^{\alpha }$ is the temporal Riemann–Liouville fractional integral operator of order *α*.

The above-mentioned problem has many physical applications in various areas. Particularly, when $\alpha =1$, problem (1.1) reduces to the classical stochastic Navier–Stokes equations, numerical approximations of which have been carried out by the authors [29, 30]. For the fractional stochastic Navier–Stokes equations, the well-posedness has been studied in [26, 27]. So far, for most fractional stochastic differential equations, it is very difficult to get exact solutions, so it is necessary to propose numerical methods. However, to the best of our knowledge, numerical analysis of such a problem for fractional stochastic Navier–Stokes equations is missing in the literature. Therefore, this article aims to fill the gap, by studying and obtaining the strong convergence approximations of fractional stochastic Navier–Stokes equations like (1.1).

In this article, our goal is to give some detailed numerical analysis of the finite element method for problem (1.1). Because the mild solution of fractional stochastic Navier–Stokes equations is provided by the solution operator $E(t)$ defined through the Mittag-Leffler function, it is different from the classic stochastic Navier–Stokes equations related to the analytic semigroup $e^{\Delta t}$. The properties of the semigroup and the semigroup theory have been studied in detail in [31, 32]. However, for the solution operator $E(t)$, as far as we are know, similar properties are less studied. The novelty of this paper is to derive the properties of the solution operator $E(t)$ which is defined through the Mittag-Leffler function and establish the Hölder regularity of the weak solutions for fractional stochastic Navier–Stokes equations. Firstly, we deduce some regularity results and stability properties of $E(t)$ which play a key role in the error analysis. The discretization in space is derived by the finite element method and the time discretization is obtained by the backward Euler scheme. Based on the error estimates for the corresponding deterministic problem and Itô isometry, finally the strong convergence error estimates for the fully discrete schemes of fractional stochastic Navier–Stokes equations are obtained.

The structure of this paper is as follows: In Sect. 2, we introduce some preliminaries and notations, as well as give the definition of the Mittag-Leffler function. In Sect. 3, we give the semidiscrete Galerkin approximations in space and then obtain the fully discrete scheme. In Sect. 4, we present several lemmas about the operator $E(t)$ which play a crucial role in the proof of the error estimate. Finally, in Sect. 5, we will give the fully discrete error estimates for the fractional stochastic Navier–Stokes equations.

## Preliminaries

Throughout the paper, we denote by *C* a constant that may not be of the same form from one occurrence to another, even in the same line. In this section, we introduce some notations and some important preliminaries.

Let $\Vert \cdot \Vert _{U}$ and $\Vert \cdot \Vert _{H}$ be the norms of separable Hilbert spaces *U* and *H*, respectively. Let $L(U,H)$ denote the space of bounded linear operators from *U* to *H*, and let $\mathcal{L}_{2}(U,H)$ be the space of Hilbert–Schmidt operators with norm

$$ \Vert T \Vert _{\mathcal{L}_{2}(U,H)}^{2}:=\sum _{k=1}^{\infty } \Vert Te_{k} \Vert _{H} ^{2}< \infty , $$

where $\{e_{k}\}_{k=1}^{\infty }$ is an orthonormal basis of *U*. If $U=H$, then $L(U)=L(U,U)$ and $HS=\mathcal{L}_{2}(U,U)$.

Let $\{\mathcal{T}_{h}\}$ be a regular family of triangulations of Ω with $h_{k}=\operatorname{diam}(K)$, $h=\max_{K\in \mathcal{T} _{h}}h_{K}$, and let $V_{h}$ denote the space of piecewise linear continuous functions with respect to $\mathcal{T}_{h}$ which vanish on *∂*Ω. Hence, $V_{h}\subset H_{0}^{1}( \Omega )=\dot{H} ^{1}=\{v\in L_{2}( \Omega ), \nabla v\in L_{2}( \Omega ), v\vert _{\partial \Omega =0}\}$. The norms in the Sobolev spaces $H^{s}( \Omega )$, $s \geq 0$, are denoted by $\Vert \cdot \Vert _{s}$. And we assume that a family $\{V_{h}\}$ of finite-dimensional subspaces of $H_{0}^{1}$ is such that, for some integer $r\geq 2$ and small *h* (cf. [31]),

2.1 $$\begin{aligned} \inf_{\chi \in V_{h}} \bigl\{ \Vert v-\chi \Vert +h \bigl\Vert \nabla (v-\chi ) \bigr\Vert \bigr\} \leq Ch^{s} \Vert v \Vert _{s}, \quad \text{for } 1\leq s\leq r, \end{aligned}$$

$v\in H^{s}\cap H_{0}^{1}$, where $H^{s}$ denotes the Sobolev space of order *s*.

Let $(\Omega , \mathcal{F}, \mathbf{P})$ be a probability space and let **E** be the expectation. For any Hilbert space, we define

$$\begin{aligned} L_{2}(\Omega ; H)= \biggl\{ v: \mathbf{E} \Vert v \Vert _{H}^{2}= \int_{\Omega } \bigl\Vert v(w) \bigr\Vert _{H}^{2} \,d\mathbf{P}(w)< \infty \biggr\} , \end{aligned}$$

with norm $\Vert v\Vert _{L_{2}(\Omega ,H)}=\mathbf{E}(\Vert v\Vert _{H}^{2})^{ \frac{1}{2}}$.

Let *Q* be the covariance operator of $W(t)$; $Q\in \mathcal{L}(U)$ is a linear, self-adjoint, positive definite, bounded operator with finite trace, i.e., $\operatorname{Tr}(Q)<\infty $, where $\operatorname{Tr}(Q)$ denotes the trace of *Q*. The stochastic process $W(t)$ is a U-valued Q-Wiener process with respect to the filtration $\{{\mathcal{F}}_{t}\}_{t \geq 0}$ if

(i) $W(0)=0$,
(ii) *W* has independent increments,
(iii) *W* has continuous trajectories (almost surely),
(iv) $W(t)-W(s)$, $0\leq s\leq t$, is a U-valued Gaussian random variable with zero mean and covariance operator $(t-s)Q$,
(v) $\{W(t)\}_{t\geq 0}$ is adapted to $\{\mathcal{F}_{t}\}$,
(vi) the random variable $W(t)-W(s)$ is independent of $\mathcal{F}_{s}$ for all fixed $s\in [0,t]$.

It is known (see, e.g., Sect. 2.1 in [33]) that for a given Q-Wiener process satisfying (i)–(iv) one can always find a normal filtration $\{{\mathcal{F}}_{t}\}_{t\geq 0}$ so that (v)–(vi) hold. Suppose that $\{(\gamma_{j},e_{j})\}_{j=1}^{\infty }$ are the eigenpairs of *Q* with orthonormal eigenvectors and $\{\beta_{j}(t)\}_{j=1}^{ \infty }$ are real-valued mutually independent standard Brownian motions. Then $W(t)$ has the orthogonal expansion

$$ W(t)=\sum_{j=1}^{\infty }\gamma_{j}^{1/2} \beta_{j}(t)e_{j}. $$

It is then possible to define the stochastic integral $\int_{0}^{t} \psi (s)\,dW(s)$ together with Itô’s isometry,

2.2 $$\begin{aligned} \mathbf{E} \biggl\Vert \int^{t}_{0}\psi (s)\,dW(s) \biggr\Vert _{H}^{2}= \int^{t}_{0}\mathbf{E} \bigl\Vert \psi (s)Q^{1/2} \bigr\Vert ^{2}_{\mathcal{L}_{2}(U,H)} \,ds. \end{aligned}$$

The operator $P_{h} : L_{2}(\Omega ) \rightarrow V_{h}$ denotes the projection operator defined by

2.3 $$\begin{aligned} (P_{h}v,\chi )=(v,\chi ),\quad v\in L_{2}(\Omega ), \forall \chi \in V_{h}. \end{aligned}$$

For the reader’s convenience, the definition of Mittag-Leffler function will be provided. We shall use the extended Mittag-Leffler function $E_{\alpha ,\beta }(z)$ [25] defined by

$$E_{\alpha ,\beta }(z)=\sum_{k=0}^{\infty }\frac{z^{k}}{\Gamma (k\alpha +\beta )},\;z\in C,$$

where $\Gamma (\cdot )$ is the standard Gamma function defined as

$$\begin{aligned} \Gamma (z)= \int_{0}^{\infty }t^{z-1}e^{-t} \,dt, \quad \Re (z)>0. \end{aligned}$$

## Discretization of fractional stochastic problem

Let Π be the divergence-free projection operator of the Helmholtz decomposition (cf., [34, 35]). In order to consider a velocity *u* satisfying *P*-a.s. (almost surely) $\nabla \cdot u=0$, we project the fractional stochastic Navier–Stokes equation onto the space of divergence-free vector fields, thereby removing the pressure $p(x,t)$. Then, applying the Helmholtz projection Π on both sides of Eq. (1.1), we obtain

3.1 $$ u_{t}+\mathscr{B}^{\alpha }Au+B(u,u)=\dot{W}, \quad \text{in } \Omega \times [0, T], $$

where $A=-\Pi \Delta $, $B(u, u) := \Pi ((u\cdot \nabla )u)$. The bilinear operator $B(\cdot , \cdot )$ satisfies the following inequality (cf., [36, 37]):

3.2 $$\begin{aligned} \bigl\Vert B \bigl(u(s), u(s) \bigr) \bigr\Vert \leq C \bigl\Vert u(s) \bigr\Vert \bigl\Vert u(s) \bigr\Vert _{1}, \end{aligned}$$

which has important applications when establishing strong convergence error estimates for the fully discrete schemes of fractional stochastic Navier–Stokes equations.

We shall assume that

$$\begin{aligned} \bigl\Vert u(s) \bigr\Vert \leq M_{1}, \quad\quad \bigl\Vert u(s) \bigr\Vert _{1}\leq M_{2},\quad 0\leq s\leq T. \end{aligned}$$

Also we assume that the operator *A* is self-adjoint and there exist eigenvectors $\varphi_{j}$ corresponding to eigenvalues $\lambda_{j}$ such that (cf., [28, 29])

$$A\varphi_{j}=\lambda_{j}\varphi_{j},\;j\in N^{+}.$$

In a standard way, the fractional powers $A^{s}$, s∈R, of *A* are introduced by

$$\begin{aligned} A^{s} v=\sum_{j=1}^{\infty } \lambda_{j}^{s}(v,\varphi_{j}) \varphi_{j}, \quad\quad D \bigl(A^{s/2} \bigr)= \Biggl\{ v\in H: \bigl\Vert A^{s/2}v \bigr\Vert ^{2}=\sum _{j=1}^{\infty } \lambda_{j}^{s}(v, \varphi_{j})^{2}< \infty \Biggr\} . \end{aligned}$$

Let $\dot{H}^{s}=D(A^{s/2})$ with its norm denoted by

3.3 $$\begin{aligned} \Vert v \Vert _{s}= \bigl\Vert A^{s/2}v \bigr\Vert = \Biggl( \sum_{j=1}^{\infty } \lambda_{j} ^{s}(v,\varphi_{j})^{2} \Biggr) ^{1/2}, \quad v\in \dot{H}^{s}. \end{aligned}$$

Now we introduce the operator $E(t)$ by

3.4 $$\begin{aligned} E(t)v=\sum_{j=1}^{\infty }E_{\alpha ,1} \bigl(-\lambda_{j} t^{\alpha } \bigr) (v,\varphi_{j}) \varphi_{j}, \quad v\in \dot{H}^{s}, \end{aligned}$$

where $\alpha \in (0,1)$ denotes the Caputo fractional derivative of order *α* and $E_{\alpha ,1}$ is the Mittag-Leffler function.

By making use of time fractional Duhamel’s priciple [38–40], the solution $u(t)$ of (3.1) at time $t=t_{n}$ can be written as

3.5 $$\begin{aligned} u(t_{n})=E(t_{n})u_{0}- \int_{0}^{t_{n}}E(t_{n}-s)B \bigl(u(s),u(s) \bigr)\,ds+ \int _{0}^{t_{n}}E(t_{n}-s)\,dW. \end{aligned}$$

Let $A_{h}: V_{h}\rightarrow V_{h}$ denote the discrete analogue of the operator *A*, i.e.,

$$\begin{aligned} (A_{h}\psi , \chi )=(\nabla \psi , \nabla \chi ),\quad \forall \psi , \chi \in V_{h}. \end{aligned}$$

Then the semidiscrete problem corresponding to (3.1) is to find the process $u_{h}(t)\in V_{h}$ such that

3.6 $$\begin{aligned} u_{ht}+\mathscr{B}^{\alpha }A_{h}u_{h}+P_{h}B(u_{h},u_{h})=P_{h} \dot{W}, \quad \text{with } u_{h}(0)=P_{h}u_{0}. \end{aligned}$$

The operator ${E}_{h}(t)$ is introduced by

3.7 $$\begin{aligned} E_{h}(t)v_{h}=\sum _{j=1}^{\infty }E_{\alpha ,1} \bigl(- \lambda_{j} ^{h} t^{\alpha } \bigr) \bigl(v, \varphi_{j}^{h} \bigr)\varphi_{j}^{h}, \quad v_{h}\in X_{h}, \end{aligned}$$

where $\{\lambda_{j}^{h}\}_{j=1}^{N}$ and $\{\varphi_{j}^{h}\}_{j=1} ^{N}$ are respectively the eigenvalues and eigenfunctions of the discrete Laplace operator $A_{h}$. Then the semidiscrete problem (3.6) has the abstract integral equation given by

$$\begin{aligned} u_{h}(t_{n})=E_{h}(t_{n})P_{h}u_{0}- \int_{0}^{t_{n}}E_{h}(t_{n}-s)P _{h}B \bigl(u_{h}(s),u_{h}(s) \bigr)\,ds+ \int_{0}^{t_{n}}E_{h}(t_{n}-s)P_{h} \,dW. \end{aligned}$$

For a fixed time step size $\Delta t>0$, we put $t_{n}=n\Delta t$ and define a piecewise-constant approximation $U_{h}^{n}\approx u(t_{n})$ by applying the DG method [41–43], namely

3.8 $$\begin{aligned}& \begin{gathered} U^{n}_{h}-U_{h}^{n-1}+ \int_{t_{n-1}}^{t_{n}}D_{t}^{1-\alpha }AU_{h}(t) \,dt+ \int_{t_{n-1}}^{t_{n}}B(U_{h},U_{h}) \,ds= \int_{t_{n-1}}^{t _{n}}\,dW \quad \text{for } n\geq 1, \\ U^{0}=P_{h}u_{0}, \end{gathered} \end{aligned}$$

where $U^{n}_{h}=U_{h}(t_{n}^{-})=\lim_{t\rightarrow t_{n}^{-}}U_{h}(t)$ denotes the one-sided limit from below at the *n*th time level. Thus, $U_{h}(t)=U_{h}^{n}$ for $t_{n-1}< t\leq t_{n}$. A short calculation shows that

$$\begin{aligned} \int_{t_{n-1}}^{t_{n}}D_{t}^{1-\alpha }AU_{h}(t) \,dt=\Delta t ^{\alpha }\sum_{j=1}^{n} \beta_{n-j}AU_{h}^{j}, \end{aligned}$$

with

$$\begin{aligned} \beta_{0}=\Delta t^{-\alpha } \int_{t_{n-1}}^{t_{n}}\frac{({t_{n}-t})^{ \alpha -1}}{\Gamma (\alpha )}\,dt= \frac{1}{\Gamma (1+\alpha )}, \end{aligned}$$

and, for $j\geq 1$,

$$\begin{aligned} \beta_{j}=\Delta t^{-\alpha } \int_{t_{n-j-1}}^{t_{n-j}}\frac{({t_{n}-t})^{ \alpha -1}-({t_{n-1}-t})^{\alpha -1}}{\Gamma (\alpha )}\,dt= \frac{(j+1)^{ \alpha }-2j^{\alpha }+(j-1)^{\alpha }}{\Gamma (1+\alpha )}. \end{aligned}$$

Then, the fully discrete mild formulation for (3.1) can be obtained as

3.9 $$\begin{aligned} U^{n}_{h}=B_{n,h}P_{h}u_{0}- \sum_{k=1}^{n} \int_{t_{k-1}}^{t _{k}}B_{n-k+1,h}P_{h}B \bigl(U_{h}^{k-1},U_{h}^{k} \bigr)\,ds+ \sum_{k=1}^{n} \int_{t_{k-1}}^{t_{k}}B_{n-k+1,h}P_{h} \,dW, \end{aligned}$$

where the detailed definition of $B_{n,h}$ can be found in [44].

## Some important lemmas for operator $E(t)$

In order to give the error estimates for the stochastic fractional problem, we will derive some lemmas for operator $E(t)$.

The following lemma presents the stability and smoothing estimate for operator $E(t)$, which play a key role in the error analysis of FEM approximations.

### Lemma 4.1

([3])

*For* $\alpha \in (0,1)$, *we have the following estimates*:

4.1 $$\begin{aligned} \bigl\Vert \bigl(D_{t}^{\alpha } \bigr)^{\ell }E(t)v \bigr\Vert _{p}\leq Ct^{-\alpha (\ell + \frac{p-q}{2})} \Vert v \Vert _{q}, \quad t>0, \end{aligned}$$

*where*, *for* $\ell =0$, $0\leq q\leq p\leq 2$, *and*, *for* $\ell =1$, $0\leq p\leq q\leq 2$ *and* $q\leq p+2$.

Next, several important properties of the Mittag-Leffler function $E_{\alpha ,\beta }(\cdot )$ will be given.

### Lemma 4.2

([45])

*For* $\lambda >0$, $\alpha >0$ *and a positive integer* m∈N, *it holds*

$$\begin{aligned} \frac{d^{m}}{dt^{m}}E_{\alpha ,1} \bigl(-\lambda t^{\alpha } \bigr)=- \lambda t ^{\alpha -m}E_{\alpha ,\alpha -m+1} \bigl(-\lambda t^{\alpha } \bigr). \end{aligned}$$

*In particular*, *if* $m=1$, *we obtain*

$$\begin{aligned} \frac{d}{dt}E_{\alpha ,1} \bigl(-\lambda t^{\alpha } \bigr)=- \lambda t^{\alpha -1}E _{\alpha ,\alpha } \bigl(-\lambda t^{\alpha } \bigr). \end{aligned}$$

The following estimates are crucial for the error analysis in the sequel.

### Lemma 4.3

*Let*

$$\begin{aligned} \overline{E}(t)v=\sum_{j=1}^{\infty }t^{\alpha -1}E_{\alpha , \alpha } \bigl(-\lambda_{j} t^{\alpha } \bigr) (v,\varphi_{j}) \varphi_{j}. \end{aligned}$$

*Then*, *for all* $t>0$, *we have*

4.2 $$\begin{aligned} \bigl\Vert \overline{E}(t)v \bigr\Vert _{p} \leq C \textstyle\begin{cases} ct^{-1+\alpha (1+\frac{q-p}{2})} \Vert v \Vert _{q}, & p-2\leq q\leq p, \\ ct^{-1+\alpha } \Vert v \Vert _{q},&p< q. \end{cases}\displaystyle \end{aligned}$$

*Besides*, *we get*

4.3 $$\begin{aligned} \frac{d}{dt}E(t)v=-A\overline{E}(t)v. \end{aligned}$$

### Proof

For the proof of (4.2), we refer to [3] and omit it here. Subsequently, we will give the detailed proof of equality (4.3). By virtue of Lemma 4.2, we have

$$\begin{aligned} \frac{d}{dt}E(t)v =&\frac{d}{dt}\sum_{j=1}^{\infty }E_{\alpha ,1} \bigl(-\lambda_{j} t^{\alpha } \bigr) (v,\varphi_{j}) \varphi_{j} \\ =&\sum_{j=1}^{\infty }(-\lambda_{j})t^{\alpha -1}E_{\alpha , \alpha } \bigl(-\lambda_{j} t^{\alpha } \bigr) (v,\varphi_{j}) \varphi_{j} \\ =&-A\overline{E}(t)v, \end{aligned}$$

which completes the proof. □

Next we will derive the properties of operator $E(t)$ which will be used throughout this paper.

### Lemma 4.4

*Let* $0\leq \mu \leq 1$, $0\leq \alpha \leq 1$. *Then there exists a constant* *C* *such that*

(i) $\Vert A^{\mu }E(t)\Vert \leq Ct^{-\mu \alpha }$.
(ii) $\Vert A^{-\mu }(E(t)-I)\Vert \leq Ct^{\mu \alpha }$.

### Proof

Firstly, we prove (i). By Lemma 4.1, with $\ell =q=0$, $p=2\mu $, one has

$$\begin{aligned} \bigl\Vert A^{\mu }E(t){\varphi } \bigr\Vert = \bigl\Vert E(t){ \varphi } \bigr\Vert _{2\mu }\leq Ct^{-\mu \alpha } \Vert {\varphi } \Vert , \end{aligned}$$

which gives

$$\begin{aligned} \bigl\Vert A^{\mu }E(t) \bigr\Vert \leq Ct^{-\mu \alpha }. \end{aligned}$$

The proof of (i) is completed.

For (ii), by making use of (4.3), we obtain

$$\begin{aligned} \bigl\Vert E(t)v-v \bigr\Vert =& \biggl\Vert \int_{0}^{t} A\overline{E}(s)v\,ds \biggr\Vert \\ =& \biggl\Vert \int_{0}^{t} A^{1-\mu }\overline{E}(s)A^{\mu }v \,ds \biggr\Vert \\ \leq & c \int_{0}^{t} s^{-1+\mu \alpha } \bigl\Vert A^{\mu }v \bigr\Vert \,ds \\ \leq & c t^{\mu \alpha } \bigl\Vert A^{\mu }v \bigr\Vert , \end{aligned}$$

the second to last inequality of which is derived from (4.2) with $p=2-2\mu $, $q=0$ in Lemma 4.3. This completes the proof of the lemma. □

In the following, the regularity of the mild solution in time will be given.

### Theorem 4.1

(Temporal regularity)

*Let* *u* *be the solution of* (3.1). *Then for* $t_{1}, t_{2} \in [0, T]$, $0\leq \mu \leq 1$, $0\leq \alpha \leq 1$, *there exists a constant* *C* *such that*

$$\begin{aligned} \bigl\Vert u(t_{1})-u(t_{2}) \bigr\Vert _{L_{2}(\Omega ;H)} \leq C(t_{1}-t_{2})^{\mu \alpha }. \end{aligned}$$

### Proof

Let $0\leq t_{1}< t_{2}\leq T$ be arbitrary. By making use of the mild solution formulation (3.5), it can be obtained that

$$\begin{aligned} u(t_{1})-u(t_{2}) =& \bigl( E(t_{1})-E(t_{2}) \bigr) u_{0} \\ & {} - \int^{t_{1}}_{0} E(t_{1}-s)B \bigl(u(s),u(s) \bigr)\,ds+ \int^{t_{2}}_{0} E(t_{2}-s)B \bigl(u(s),u(s) \bigr)\,ds \\ & {} + \int^{t_{1}}_{0} E(t_{1}-s)\,dW- \int^{t_{2}}_{0} E(t_{2}-s)\,dW \\ =&L_{1}+L_{2}+L_{3}, \end{aligned}$$

where

$$\begin{aligned}& L_{1}= \bigl( E(t_{1})-E(t_{2}) \bigr) u_{0}, \\& L_{2}=- \int^{t_{1}}_{0}E(t_{1}-s)B \bigl(u(s),u(s) \bigr)\,ds+ \int^{t_{2}}_{0}E(t _{2}-s)B \bigl(u(s),u(s) \bigr)\,ds, \\& L_{3}= \int^{t_{1}}_{0} E(t_{1}-s)\,dW- \int^{t_{2}}_{0} E(t_{2}-s)\,dW. \end{aligned}$$

In the sequel, each term will be estimated separately.

For the first term $L_{1}$, by virtue of Lemmas 4.3 and 4.4, one has

4.4 $$\begin{aligned} \Vert L_{1} \Vert _{L_{2}(\Omega ;H)} =& \bigl\Vert \bigl( E(t_{1})-E(t_{2}) \bigr) u_{0} \bigr\Vert _{L_{2}(\Omega ;H)} \\ = &{ \biggl\Vert - \int_{t_{1}}^{t_{2}}E'(s)dsu_{0} \biggr\Vert _{L_{2}(\Omega ;H)}} \\ = &{ \biggl\Vert \int_{t_{1}}^{t_{2}}A\overline{E}(s)dsu_{0} \biggr\Vert _{L_{2}(\Omega ;H)}} \\ = &{ \biggl\Vert \int_{t_{1}}^{t_{2}}A^{1-\mu }\overline{E}(s)dsA^{\mu }u_{0} \biggr\Vert _{L_{2}(\Omega ;H)}} \\ \leq & C \vert t_{1}-t_{2} \vert ^{\mu \alpha } \Vert u_{0} \Vert _{L_{2}(\Omega ;H^{2 \mu })} \\ \leq & C \vert t_{1}-t_{2} \vert ^{\mu \alpha }. \end{aligned}$$

The second term $L_{2}$ can be split into two terms:

$$\begin{aligned} L_{2} =&- \int^{t_{2}}_{0} \bigl( E(t_{1}-s)-E(t_{2}-s) \bigr) B \bigl(u(s),u(s) \bigr)\,ds- \int_{t_{2}}^{t_{1}}E(t_{1}-s)B \bigl(u(s),u(s) \bigr)\,ds \\ =&L_{21}+L_{22}, \end{aligned}$$

where $L_{21}$ and $L_{22}$ are estimated as follows.

For $L_{21}$, by making use of Lemma 4.4, as well as property (3.2) of $B(\cdot , \cdot )$,

4.5 $$\begin{aligned} \Vert L_{21} \Vert _{L_{2}(\Omega ;H)} =& \biggl\Vert \int^{t_{2}}_{0} \bigl( E(t_{1}-s)-E(t _{2}-s) \bigr) B \bigl(u(s),u(s) \bigr)\,ds \biggr\Vert _{L_{2}(\Omega ;H)} \\ = &{ \biggl\Vert \int^{t_{2}}_{0} \int_{t_{1}}^{t_{2}}-E'(\tau -s)B \bigl(u(s),u(s) \bigr)\,d\tau \,ds \biggr\Vert _{L_{2}(\Omega ;H)}} \\ = &{ \biggl\Vert \int^{t_{2}}_{0} \int_{t_{1}}^{t_{2}}A\overline{E}(\tau -s)B \bigl(u(s),u(s) \bigr)\,d \tau \,ds \biggr\Vert _{L_{2}(\Omega ;H)}} \\ = &{ \biggl\Vert \int^{t_{2}}_{0} \int_{t_{1}}^{t_{2}}A^{1-\mu } \overline{E}(\tau -s)A^{\mu }B \bigl(u(s),u(s) \bigr)\,d\tau \,ds \biggr\Vert _{L_{2}( \Omega ;H)}} \\ \leq & C(t_{1}-t_{2})^{\mu \alpha }. \end{aligned}$$

By Lemma 4.4,

4.6 $$\begin{aligned} \Vert L_{22} \Vert _{L_{2}(\Omega ;H)} =& \biggl\Vert \int_{t_{2}}^{t_{1}}E(t_{1}-s)B \bigl(u(s),u(s) \bigr)\,ds \biggr\Vert _{L_{2}(\Omega ;H)} \\ \leq & C(t_{1}-t_{2})^{\mu \alpha }. \end{aligned}$$

Similarly, the third term $L_{3}$ can be written as

$$\begin{aligned} L_{3} =& \int^{t_{2}}_{0} \bigl( E(t_{1}-s)-E(t_{2}-s) \bigr) \,dW+ \int ^{t_{1}}_{t_{2}} E(t_{1}-s)\,dW \\ =&L_{31}+L_{32}. \end{aligned}$$

By making use of Itô’s isometry and Lemma 4.4, it can be deduced that

4.7 $$\begin{aligned} \Vert L_{31} \Vert ^{2}_{L_{2}(\Omega ;H)} =& \biggl\Vert \int^{t_{2}}_{0} \bigl( E(t_{1}-s)-E(t _{2}-s) \bigr) \,dW \biggr\Vert ^{2}_{L_{2}(\Omega ;H)} \\ \leq & \int^{t_{2}}_{0} \bigl\Vert E(t_{1}-s)-E(t_{2}-s) \bigr\Vert ^{2}\,ds \\ \leq & C(t_{1}-t_{2})^{2\mu \alpha }. \end{aligned}$$

The term $L_{32}$ is estimated analogously by using Lemma 4.4, namely

4.8 $$\begin{aligned} \Vert L_{32} \Vert _{L_{2}(\Omega ;H)}\leq C(t_{1}-t_{2})^{\mu \alpha }. \end{aligned}$$

Combining (4.4)–(4.8) yields the result. □

## Error estimates for the stochastic fractional N–S equations

In this section, we will give the fully discrete error estimates for the stochastic fractional Navier–Stokes equations.

Let $e^{n}=U^{n}_{h}-u(t_{n})$. Then, by (3.9) and (3.5), it can be obtained that

$$\begin{aligned} e^{n} =& \bigl[B_{n,h}P_{h}-E(t_{n}) \bigr]u_{0} \\ & {} + \int^{t_{n}}_{0} E(t_{n}-s)B(u,u)\,ds-\sum _{k=1}^{n} \int_{t _{k-1}}^{t_{k}}B_{n-k+1,h}P_{h}B \bigl(U_{h}^{k-1},U_{h}^{k} \bigr)\,ds \\ & {} +\sum_{k=1}^{n} \int_{t_{k-1}}^{t_{k}}B_{n-k+1,h}P_{h} \,dW- \int_{0}^{t_{n}}E(t_{n}-s)\,dW \\ =:&I+\mathit{II}+\mathit{III}, \end{aligned}$$

where

$$\begin{aligned}& I= \bigl[B_{n,h}P_{h}-E(t_{n}) \bigr]u_{0}, \\& \mathit{II}= \int^{t_{n}}_{0} E(t_{n}-s)B(u,u)\,ds-\sum _{k=1}^{n} \int_{t _{k-1}}^{t_{k}}B_{n-k+1,h}P_{h}B \bigl(U_{h}^{k-1},U_{h}^{k} \bigr)\,ds, \\& \mathit{III}=\sum_{k=1}^{n} \int_{t_{k-1}}^{t_{k}}B_{n-k+1,h}P_{h} \,dW- \int_{0}^{t_{n}}E(t_{n}-s)\,dW. \end{aligned}$$

Next, each term will be estimated in turn.

In order to prove the main error estimates, we need the following useful conclusions for the corresponding deterministic problem, see [44] for more details.

### Lemma 5.1

([44])

*Let* $0\leq \beta \leq 2$, $F_{n,h}=B_{n,h}P_{h}-E(t_{n})$. *Then*

$$\begin{aligned} \Vert F_{n,h} \Vert \leq C \bigl(h^{\beta }+k \bigr). \end{aligned}$$

The following lemma is the time discrete version with smooth initial data.

### Lemma 5.2

([43])

*Let* $U^{n}=B_{n,h}P_{h} u_{0}$, $u(t_{n})=E_{h}(t)P_{h}u_{0}$. *Then*

$$\begin{aligned} \bigl\Vert U^{n}-u(t_{n}) \bigr\Vert \leq Ct_{n}^{r\alpha -1}\Delta t \bigl\Vert A^{r}u_{0} \bigr\Vert , \quad 0\leq r\leq \min \{2,1/\alpha \}. \end{aligned}$$

### Remark 5.1

From the above lemma, it is not difficult to find that

$$\begin{aligned} \Vert B_{n,h} \Vert \leq C, \quad \text{for all } n\geq 1, h>0. \end{aligned}$$

Firstly, we derive the error estimate of the second term *II* of the main error $e^{n}$.

### Lemma 5.3

*Let* *II* *be defined as above*. *For* $0<\mu <1$, $0<\alpha <1$, $0\leq \beta \leq 2$, *there exists a constant* *C* *such that*

$$\begin{aligned} \Vert \mathit{II} \Vert _{L_{2}(\Omega ;H)}\leq Ck^{\mu \alpha }+C \bigl(h^{\beta }+k \bigr)+Ck \sum_{k=1}^{n} \bigl\Vert e^{k-1} \bigr\Vert ^{2}_{1}. \end{aligned}$$

### Proof

The second term *II* can be split into the following five terms, and each term will be estimated separately.

$$\begin{aligned} \mathit{II} & = \int^{t_{n}}_{0} E(t_{n}-s)B \bigl(u(s),u(s) \bigr)\,ds-\sum_{k=1}^{n} \int_{t_{k-1}}^{t_{k}}B_{n-k+1,h}P_{h}B \bigl(U_{h}^{k-1},U_{h}^{k} \bigr)\,ds \\ & =\sum_{k=1}^{n} \int_{t_{k-1}}^{t_{k}}E(t_{n}-s)B \bigl(u(s),u(s) \bigr)\,ds- \sum_{k=1}^{n} \int_{t_{k-1}}^{t_{k}}E(t_{n}-s)B \bigl(u(t_{k}),u(t _{k}) \bigr)\,ds \\ & \quad {} +\sum_{k=1}^{n} \int_{t_{k-1}}^{t_{k}}E(t_{n}-s)B \bigl(u(t_{k}),u(t _{k}) \bigr)\,ds-\sum _{k=1}^{n} \int_{t_{k-1}}^{t_{k}}E(t_{n}-t_{k-1})B \bigl(u(t _{k}),u(t_{k}) \bigr)\,ds \\ & \quad {} +\sum_{k=1}^{n} \int_{t_{k-1}}^{t_{k}}E(t_{n}-t_{k-1})B \bigl(u(t _{k}),u(t_{k}) \bigr)\,ds-\sum _{k=1}^{n} \int_{t_{k-1}}^{t_{k}}B_{n-k+1,h}P _{h}B \bigl(u(t_{k}),u(t_{k}) \bigr)\,ds \\ & \quad {} +\sum_{k=1}^{n} \int_{t_{k-1}}^{t_{k}}B_{n-k+1,h}P_{h}B \bigl(u(t _{k}),u(t_{k}) \bigr)\,ds-\sum _{k=1}^{n} \int_{t_{k-1}}^{t_{k}}B_{n-k+1,h}P _{h}B \bigl(u(t_{k-1}),u(t_{k}) \bigr)\,ds \\ & \quad {} +\sum_{k=1}^{n} \int_{t_{k-1}}^{t_{k}}B_{n-k+1,h}P_{h}B \bigl(u(t _{k-1}),u(t_{k}) \bigr)\,ds-\sum _{k=1}^{n} \int_{t_{k-1}}^{t_{k}}B_{n-k+1,h}P _{h}B \bigl(U_{h}^{k-1},U_{h}^{k} \bigr)\,ds \\ & =:\mathit{II}_{1}+\mathit{II}_{2}+ \mathit{II}_{3}+\mathit{II}_{4}+\mathit{II}_{5}. \end{aligned}$$

The term $\mathit{II}_{1}$ is estimated by applying Lemma 4.4 and Theorem 4.1, which yield

5.1 $$\begin{aligned} \Vert \mathit{II}_{1} \Vert _{L_{2}(\Omega ;H)} =& \Biggl\Vert \sum_{k=1}^{n} \int_{t_{k-1}} ^{t_{k}}E(t_{n}-s) \bigl[B \bigl(u(s),u(s) \bigr)-B \bigl(u(t_{k}),u(t_{k}) \bigr) \bigr]\,ds \Biggr\Vert _{L_{2}( \Omega ;H)} \\ \leq & C\sum_{k=1}^{n} \int_{t_{k-1}}^{t_{k}}\Vert B(u(s),u(s)-B \bigl(u(t _{k}),u(t_{k}) \bigr)\Vert \,ds \\ \leq & C\sum_{k=1}^{n} \int_{t_{k-1}}^{t_{k}}(\Vert B \bigl(u(s),u(s)-B \bigl(u(t _{k}),u(s) \bigr) \Vert \\ & {} + \bigl\Vert B \bigl(u(t_{k}),u(s) \bigr)-B \bigl(u(t_{k}),u(t_{k}) \bigr) \bigr\Vert \bigr)\,ds \\ \leq & C\sum_{k=1}^{n} \int_{t_{k-1}}^{t_{k}}(s-t_{k})^{\mu \alpha }\,ds \\ \leq & Ck^{\mu \alpha }. \end{aligned}$$

For the term $\mathit{II}_{2}$, by making use of Lemma 4.4 and property (3.2) of $B(\cdot , \cdot )$, one can arrive at

5.2 $$\begin{aligned} \Vert \mathit{II}_{2} \Vert _{L_{2}(\Omega ;H)} =& \Vert \sum_{k=1}^{n} \int_{t_{k-1}} ^{t_{k}} \bigl(E(t_{n}-s)-E(t_{n}-t_{k-1}) \bigr)B \bigl(u(t_{k}),u(t_{k}) \bigr)\,ds \\ \leq &C\sum_{k=1}^{n} \int_{t_{k-1}}^{t_{k}} \bigl\Vert \bigl(E(t_{n}-s)-E(t _{n}-t_{k-1}) \bigr) \bigr\Vert \,ds \\ \leq & Ck^{\mu \alpha }. \end{aligned}$$

The estimate for $\mathit{II}_{3}$ is a straightforward application of Lemma 5.1 and property (3.2) of $B(\cdot , \cdot )$ yielding

5.3 $$\begin{aligned} \Vert \mathit{II}_{3} \Vert _{L_{2}(\Omega ;H)} =& \sum_{k=1}^{n} \int_{t_{k-1}} ^{t_{k}} \bigl(E(t_{n}-t_{k-1})-B_{n-k+1,h}P_{h} \bigr)B \bigl(u(t_{k}),u(t_{k}) \bigr)\,ds \\ \leq & C\sum_{k=1}^{n} \int_{t_{k-1}}^{t_{k}} \bigl(h^{\beta }+k \bigr)\,ds \\ \leq & C \bigl(h^{\beta }+k \bigr). \end{aligned}$$

For the term $\mathit{II}_{4}$, by virtue of the property of $B_{n-k+1,h}$ in Lemma 5.2 and Theorem 4.1, there holds

5.4 $$\begin{aligned} \Vert \mathit{II}_{4} \Vert _{L_{2}(\Omega ;H)} =& \Biggl\Vert \sum_{k=1}^{n} \int_{t_{k-1}} ^{t_{k}}B_{n-k+1,h}P_{h} \bigl(B \bigl(u(t_{k}),u(t_{k}) \bigr)-B \bigl(u(t_{k-1}),u(t_{k}) \bigr) \bigr)\,ds \Biggr\Vert \\ \leq & Ck. \end{aligned}$$

The term $\mathit{II}_{5}$ is similarly bounded by the property of $B_{n-k+1,h}$ in Lemma 5.2, namely

5.5 $$\begin{aligned} \Vert \mathit{II}_{5} \Vert _{L_{2}(\Omega ;H)} =& \Biggl\Vert \sum_{k=1}^{n} \int_{t_{k-1}} ^{t_{k}}B_{n-k+1,h}P_{h} \bigl(B \bigl(u(t_{k-1}),u(t_{k}) \bigr)-B \bigl(U_{h}^{k-1},U_{h} ^{k} \bigr) \bigr)\,ds \Biggr\Vert _{L_{2}(\Omega ;H)} \\ \leq & Ck\sum_{k=1}^{n} \bigl\Vert e^{k-1} \bigr\Vert ^{2}_{1}. \end{aligned}$$

Due to (5.1)–(5.5), we complete the proof. □

Similarly, we consider the error estimate of the third term *III*.

### Lemma 5.4

*Let* *III* *be defined as above*. *For* $0<\mu <1$, $0<\alpha <1$, $0\leq \beta \leq 2$, *there exists a constant* *C* *such that*

$$\begin{aligned} \Vert \mathit{III} \Vert _{L_{2}(\Omega ;H)}\leq C \bigl(h^{\beta }+k^{\mu \alpha } \bigr). \end{aligned}$$

### Proof

The term *III* can be split into the following terms:

$$\begin{aligned} \mathit{III} =&\sum_{k=1}^{n} \int_{t_{k-1}}^{t_{k}}B_{n-k+1,h}P_{h} \,dW- \int_{0}^{t_{n}}E(t_{n}-s)\,dW \\ =&\sum_{k=1}^{n} \int_{t_{k-1}}^{t_{k}} \bigl(B_{n-k+1,h}P_{h}-E(t _{n}-t_{k-1}) \bigr)\,dW \\ &{} +\sum_{k=1}^{n} \int_{t_{k-1}}^{t_{k}} \bigl(E(t_{n}-t_{k-1})-E(t _{n}-s) \bigr)\,dW \\ =:&\mathit{III}_{1}+\mathit{III}_{2}. \end{aligned}$$

For the term $\mathit{III}_{1}$, by virtue of Itô’s isometry and Lemma 5.1, it holds

5.6 $$\begin{aligned} \Vert \mathit{III}_{1} \Vert ^{2}_{L_{2}(\Omega ;H)} \leq & C\sum_{k=1}^{n} \int_{t_{k-1}}^{t_{k}} \bigl\Vert \bigl(B_{n-k+1,h}P_{h}-E(t_{n}-t_{k-1}) \bigr) \bigr\Vert ^{2}\,ds \\ \leq & C \bigl(h^{2\beta }+k^{2} \bigr). \end{aligned}$$

By Itô’s isometry and Lemma 4.4, the estimate for $\mathit{III}_{2}$ is obtained as follows:

5.7 $$\begin{aligned} \Vert \mathit{III}_{2} \Vert ^{2}_{L_{2}(\Omega ;H)} =& \Biggl\Vert \sum_{k=1}^{n} \int_{t _{k-1}}^{t_{k}} \bigl(E(t_{n}-t_{k-1})-E(t_{n}-s) \bigr)\,dW \Biggr\Vert ^{2} \\ =&\sum_{k=1}^{n} \int_{t_{k-1}}^{t_{k}} \bigl\Vert \bigl(E(t_{n}-t_{k-1})-E(t _{n}-s) \bigr) \bigr\Vert ^{2}\,ds \\ \leq & {Ck^{2\mu \alpha }}. \end{aligned}$$

Hence, by (5.6) and (5.7), the proof is completed. □

Based on the above conclusions, the main theorem of the paper can now be obtained.

### Theorem 5.1

*Let* $0\leq \beta \leq 2$, $0\leq \mu \leq 1$, $0\leq \alpha \leq 1$. *Then*

$$\begin{aligned} \bigl\Vert U^{n}_{h}-u(t_{n}) \bigr\Vert _{L_{2}(\Omega ;H)}\leq C \bigl(h^{\beta }+k^{\mu \alpha } \bigr). \end{aligned}$$

### Proof

First of all, for the term *I*, by applying Lemma 5.1, it can be obtained that

5.8 $$\begin{aligned} \Vert I \Vert _{L_{2}(\Omega ;H)}\leq C \bigl(h^{\beta }+k^{\mu \alpha } \bigr). \end{aligned}$$

Combining with (5.8), Lemma 5.3, Lemma 5.4, we conclude that

$$\begin{aligned} \bigl\Vert e^{n} \bigr\Vert _{L_{2}(\Omega ;H)}\leq Ck^{\mu \alpha }+C \bigl(h^{\beta }+k \bigr)+Ck \sum _{k=1}^{n} \bigl\Vert e^{k-1} \bigr\Vert ^{2}_{1}, \end{aligned}$$

by using the discrete Gronwall’s lemma, this yields

$$\begin{aligned} \bigl\Vert U^{n}_{h}-u(t_{n}) \bigr\Vert _{L_{2}(\Omega ;H)}\leq C \bigl(h^{\beta }+k^{\mu \alpha } \bigr), \end{aligned}$$

which completes the proof. □
